# Population Genetic Structure of *Streptococcus pneumoniae* in Kilifi, Kenya, Prior to the Introduction of Pneumococcal Conjugate Vaccine

**DOI:** 10.1371/journal.pone.0081539

**Published:** 2013-11-25

**Authors:** Angela B. Brueggemann, Beth Mbesu Muroki, Benard W. Kulohoma, Angela Karani, Eva Wanjiru, Susan Morpeth, Tatu Kamau, Shahnaaz Sharif, J. Anthony G. Scott

**Affiliations:** 1 Department of Zoology, University of Oxford, Oxford, United Kingdom; 2 Nuffield Department of Medicine, University of Oxford, Oxford, United Kingdom; 3 KEMRI-Wellcome Trust Research Programme, Kilifi, Kenya; 4 Ministry of Public Health and Sanitation, Nairobi, Kenya; Centers for Disease Control & Prevention, United States of America

## Abstract

**Background:**

The 10-valent pneumococcal conjugate vaccine (PCV10) was introduced in Kenya in 2011. Introduction of any PCV will perturb the existing pneumococcal population structure, thus the aim was to genotype pneumococci collected in Kilifi before PCV10.

**Methods and Findings:**

Using multilocus sequence typing (MLST), we genotyped >1100 invasive and carriage pneumococci from children, the largest collection genotyped from a single resource-poor country and reported to date. Serotype 1 was the most common serotype causing invasive disease and was rarely detected in carriage; all serotype 1 isolates were members of clonal complex (CC) 217. There were temporal fluctuations in the major circulating sequence types (STs); and although 1-3 major serotype 1, 14 or 23F STs co-circulated annually, the two major serotype 5 STs mainly circulated independently. Major STs/CCs also included isolates of serotypes 3, 12F, 18C and 19A and each shared ≤2 MLST alleles with STs that circulate widely elsewhere. Major CCs associated with non-PCV10 serotypes were predominantly represented by carriage isolates, although serotype 19A and 12F CCs were largely invasive and a serotype 10A CC was equally represented by invasive and carriage isolates.

**Conclusions:**

Understanding the pre-PCV10 population genetic structure in Kilifi will allow for the detection of changes in prevalence of the circulating genotypes and evidence for capsular switching post-vaccine implementation.

## Introduction

The introduction of pneumococcal conjugate vaccine (PCV) into immunisation programmes in well-resourced countries led to a significant reduction in pneumococcal morbidity and mortality [1,2]. Children in resource-poor countries have a much higher incidence of life-threatening pneumococcal disease [3,4], thus the World Health Organisation recommended that PCV be introduced into developing countries with high childhood mortality, and the GAVI Alliance has provided support for PCV introduction [5]. Pneumococcal disease burden among young children living within the Kilifi District on the coast of Kenya is very high: the annual incidence of clinically-significant pneumococcal bacteraemia among children <5 years of age who presented to the outpatient department of Kilifi District Hospital was estimated at 436 cases per 100,000 [6]; and among children who were <1, <2 and <5 years of age and admitted to hospital the incidence was 241, 213 and 111 cases per 100,000, respectively [7]. Nasopharyngeal carriage prevalence among healthy children is also high: the overall population-based prevalence in a pre-PCV10 study was 66% (79% for children <1 year of age; 51% among children 4.5-5.0 yrs of age) [8]. Therefore, in January 2011, Kenya introduced the 10-valent PCV (PCV10), which contains serotypes 1, 4, 5, 6B, 7F, 9V, 14, 18C, 19F and 23F, into its childhood immunisation programme. PCV10 coverage was estimated to be 42% among carriage isolates and >70% among invasive pneumococci [8,9]. In January and March 2011 the Kenyan Government conducted a two-dose catch-up campaign for all children aged <5 years in Kilifi District to accelerate the conditions of a mature vaccination programme. Population-based surveillance established in 2001 at Kilifi District Hospital will be used to evaluate the impact of PCV10 introduction. 

In well-resourced countries, PCV introduction led to a significant overall reduction in the incidence of invasive pneumococcal disease due to vaccine serotypes. PCV also led to a profound reduction in the prevalence of nasopharyngeal carriage of vaccine serotypes among healthy children, but with a compensatory rise in the prevalence of nonvaccine serotypes. This resulted in fundamental changes in the transmission patterns of serotypes, which had an effect on disease: the reduction in transmission of vaccine serotype pneumococci led to a herd-protection effect, benefiting older unvaccinated individuals, and the increased circulation of nonvaccine serotypes led to ‘serotype replacement disease’, which attenuated the net benefit of PCV introduction. However, many nonvaccine serotypes appear to be inherently less invasive than vaccine serotypes, thus the reduction in invasive disease caused by vaccine serotypes has, in most populations, exceeded the increase in serotype replacement disease [10-18]. Changes in the circulating serotypes also resulted in a concomitant change in the circulating genotypes in developed countries, since the serotype and multilocus sequence typing (MLST) genotype are closely associated, with known exceptions [14,19-22]. Much of the focus centred around changes in the prevalence of serotype 19A-associated genotypes, since 19A was the predominant non-vaccine serotype that increased in prevalence after PCV7 introduction, but there were changes in the prevalence of other genotypes as well [13,23,24]. 

In resource-poor countries, much less is known about which serotypes and genotypes are circulating at a population level, thus the impact of PCV introduction on the pneumococcal population structure and the potential for evolution in response to vaccine selective pressure cannot easily be predicted based on the experience of introducing PCV to well-resourced countries. Therefore, in this study we used MLST to provide the first large-scale characterisation of >1100 invasive and colonising pneumococcal isolates from a single resource-poor country, with the aim of revealing the population structure prior to PCV10 introduction.

## Methods

### Ethical Statement

The project, SSC#1357, entitled “The effect of routine immunization with Pneumococcal Conjugate Vaccine in children on the strain structure of invasive and carriage isolates of *S. pneumoniae* in children and adults in Kilifi District”, was reviewed by the KEMRI National Ethical Review Committee and they approved the analysis of pneumococcal isolates collected though routine surveillance at Kilifi District Hospital. They also approved the analysis of carriage isolates collected in specific research studies; for these studies individual informed consent was obtained from every parent/guardian. All data in this genotyping study were analysed anonymously. 

### Selection of Isolates for Genotyping

Invasive isolates were recovered from the blood, cerebrospinal fluid or pleural fluid of ill children <15 years of age from 1994-2008 (total n = 628). All available unique patient isolates from 1994-2002 were included and isolates were systematically selected (e.g. every other isolate in the line listing of isolates) from years 2003-2008 to obtain 25 or 50 unique patient isolates in each year’s sample for genotyping. Three isolate samples from 2003-2008 were mixed or nonviable and were not genotyped. 

Carriage isolates (n = 486) were collected during two previous studies conducted in the Kilifi District. The first study was performed in 2004 and sampled the nasopharynx of healthy persons of all age groups [25]; all available unique patient isolates recovered from children <5 yrs of age (n = 170) were included in the current genotyping study. The second study was performed from 2006-2008 and sampled healthy children <5 years of age in a rolling cross-sectional study of nasopharyngeal carriage; 320 isolates were randomly selected for genotyping from the entire collection of 1868 isolates, 4 of which were later removed as freezer stocks were nonviable [8]. 

In all studies, samples were cultured and pneumococci identified using standard microbiological methods. Isolates were serogrouped by latex agglutination and serotyped using the Quellung method in the Kilifi Laboratory. All invasive isolates were also serotyped by PCR using a published protocol, modified to better suit the Kilifi serotype distribution [26]. Any discrepancies that arose between the Quellung and PCR results were retested until consensus was achieved and the consensus serotypes were used in this study.

### MLST, Data Confirmation and Analyses

MLST was performed according to the S. *pneumoniae* MLST protocol [27] and alleles and sequence types (STs) were assigned using the MLST website [19]. Unusual combinations of serotype and genotype were verified by repeating the genotyping and/or confirming the serotype. Serotype confirmation was done either by repeat testing in Kilifi or by PCR serotyping in Oxford using PCR serotyping primers and protocols adapted from previously published methods [26,28]. We were unable to PCR-amplify the *gdh* locus for one invasive serotype 23F isolate and the *ddl* locus for one carriage serotype 19F isolate despite repeated attempts and redesigned primers (presumably due to divergent sequence in the primer binding regions), so these two isolates were removed from genotyping analyses. The data were compiled and analysed using Microsoft Excel and STATA v. 11. STs were clustered into clonal complexes (CCs) using Phyloviz [29] with the following settings: Dataset type, Multi-locus Sequence Typing; Distance, eBURST Distance; and Level, SLV. The Kilifi dataset was combined with the entire MLST database (as of February 2012; total combined n = 16,070 isolates) for the Phyloviz analyses. The entire Kilifi dataset was also submitted to the MLST database.

## Results

### Serotype and Sequence Type Distributions and Identification of Clonal Complexes

1114 invasive and carriage isolates were recovered from children in Kilifi from 1994-2008 and genotyped by MLST (Table 1). Isolate representatives of all PCV10 serotypes were found; 8 of the 10 most abundant serotypes in the invasive collection were PCV10 serotypes, as were 5 of the 10 most common serotypes in the carriage collection. Although serotype 7F was a minor serotype in this collection (n = 3 isolates), all other PCV10 serotypes were well represented (29-161 isolates characterised per serotype). Vaccine serotypes 1, 6B, 14 and 19F were each represented by >100 isolates. 

**Table 1:** Serotype distributions among the invasive (1994-2008) and carriage (2004, 2006-2008) collections of pneumococci that were genotyped by MLST.

	All	Invasive isolates					Carriage isolates		
Serotype**^*a*^**	isolates	0-1y	2-4y	5-14y	All ages		0-1y	2-4y	All ages
**1**	161	33	37	88	158		0	3	3
**6B**	107	37	13	6	56		25	26	51
**19F**	104	17	4	4	25		38	41	79
**14**	103	51	12	13	76		15	12	27
6A	93	28	7	6	41		25	27	52
**23F**	86	33	8	4	45		19	22	41
**5**	45	30	5	10	45		0	0	0
**18C**	39	19	7	6	32		1	6	7
**9V**	31	12	2	2	16		5	10	15
**4**	29	8	4	11	23		2	4	6
19A	28	12	5	2	19		7	2	9
35B	26	6	0	0	6		9	11	20
15B/C	23	2	1	0	3		7	13	20
3	20	8	1	6	15		3	2	5
11A	20	2	1	0	3		7	10	17
10A	19	6	0	3	9		6	4	10
23B	18	2	0	0	2		3	13	16
12F	16	5	2	7	14		1	1	2
15A	16	2	3	0	5		7	4	11
13	15	2	0	0	2		6	7	13
34	11	3	0	0	3		3	5	8
16F	9	2	0	1	3		3	3	6
7C	9	0	1	0	1		4	4	8
19B	8	0	1	0	1		3	4	7
20	7	0	0	0	0		1	6	7
21	6	1	0	0	1		1	4	5
24F	7	2	1	1	4		0	3	3
23A	5	0	0	0	0		2	3	5
35A	5	0	0	0	0		2	3	5
29	4	2	0	1	3		1	0	1
38	4	1	1	0	2		1	1	2
10F	4	2	1	0	3		0	1	1
17F	4	0	0	0	0		2	2	4
10B	3	0	0	0	0		0	3	3
33B	3	1	0	0	1		1	1	2
**7F**	3	3	0	0	3		0	0	0
9L	3	0	0	2	2		0	1	1
8	2	0	0	0	0		1	1	2
37	2	0	0	0	0		1	1	2
18F	2	2	0	0	2		0	0	0
28F	2	1	0	0	1		1	0	1
33D	2	0	0	0	0		2	0	2
35F	2	0	0	0	0		0	2	2
2	1	1	0	0	1		0	0	0
18	1	0	0	0	0		0	1	1
31	1	0	0	0	0		0	1	1
15F	1	0	0	0	0		0	1	1
18B	1	1	0	0	1		0	0	0
22A	1	1	0	0	1		0	0	0
9N	1	0	0	0	0		1	0	1
nontypeable	1	0	0	0	0		1	0	1
Total	1114	338	117	173	628		217	269	486

a Serotypes in boldface font are included in PCV10. Invasive isolates were serotyped by both Quellung and PCR; discordant results were generally at the level of serotype (e.g. 6A vs. 6B). Testing was repeated until consensus was achieved and the consensus serotypes were used in this study.

There were 21 major (>15 isolate representatives) CCs identified among the entire collection of pneumococci, 13 of which were of PCV10 serotypes and 8 of which were found among non-PCV10 serotypes (Tables 2 and 3). An initial observation was that the STs and CCs circulating in Kilifi were largely different to those found circulating among most developed countries (as reported to the MLST database [19]). We submitted 40 novel alleles and 212 novel STs to the MLST database; the novel STs described 26.3% of the invasive isolates and 30.9% of the carriage isolates. The clonal complexes and sequence types found among the invasive and carriage collections, stratified by serotype, are described in Tables S1 and S2, respectively. 

**Table 2:** Major clonal complexes^a^ identified among isolates of PCV10 serotypes.

CC	ST	Total	Invasive	Carriage	Predominant serotype	Other serotype(s)
217^1^	--	161	158	3	1 (100%)	-
	217	106	104	2	1	-
	613	27	26	1	1	-
	614	26	26	0	1	-
	Other	2	2	0	1	-
246^4^	--	24	20	4	4 (95.8%)	2
	853	20	17	3	4	-
	Other	4	3	1	4 (75.0%)	2
289^5^	--	44	44	0	5 (100%)	-
	245	26	26	0	5	-
	289	14	14	0	5	-
	Other	4	4	0	5	-
706^9^V	--	17	5	12	9V (100%)	-
	706	7	2	5	9V	-
	5283	6	2	4	9V	-
	Other	4	1	3	9V	-
1381^18^C	--	35	28	7	18C (100%)	-
	1381	31	24	7	18C	-
	Other	4	4	0	18C (75.0%)	18B
854^6^B	--	30	13	17	6B (86.7%)	6A
	854	28	13	15	6B (85.7%)	6A
	Other	2	0	2	6B	-
2713^6^B	--	24	17	7	6B (91.7%)	6A, 23F
	2713	10	7	3	6B	-
	5302	6	6	0	6B	-
	Other	8	4	4	6B (75.0%)	6A, 23F
63^14^	--	61	42	19	14 (100%)	-
	842	44	27	17	14	-
	2716	5	5	0	14	-
	Other	12	10	2	14	-
230^14,3^	--	40	31	9	14 (67.5%)	3
	230	24	20	4	14	-
	700	11	8	3	3	-
	Other	5	3	2	14 (60.0%)	3
844^19^F	--	50	10	40	19F (92.0%)	11A, 14, 23A, 23F
	844	19	6	13	19F (94.7%)	14
	5339	15	2	13	19F (80.0%)	11A, 23A, 23F
	5367	5	--	5	19F	-
	Other	11	2	9	19F	-
2715^19^F	--	21	9	12	19F (95.2%)	14
	2715	11	4	7	19F	-
	6088	6	4	2	19F (83.3%)	14
	Other	4	1	3	19F	-
2714^23^F	--	43	23	20	23F (100%)	-
	2714	39	22	17	23F	-
	Other	4	1	3	23F	-
988^23^F	--	25	16	9	23F (88.0%)	4, 14, 19F, 35B
	988	17	10	7	23F (94.1%)	19F
	Other	8	6	2	23F (62.5%)	4, 14, 35B

Note: CC = clonal complex; ST = sequence type; ‘-’ indicates that no other serotypes were detected.

a CCs with >15 isolate representatives shown here; STs within a CC that have fewer than 5 isolate representatives are grouped as “Other”.

**Table 3:** Major clonal complexes^a^ identified among isolates of non-PCV10 serotypes.

CC	ST	Total	Invasive	Carriage	Predominant serotype(s)	Other serotypes
701^13,15BC^	--	31	5	26	13 (48.4%), 15BC (16.1%)	6B, 9V, 23F
	701	18	5	13	13 (61.1%), 6B (16.7%)	9V (11.1%), 15BC (11.1%)
	5340	6	--	6	15BC	-
	Other	7	0	7	13 (57.1%), 15BC (28.6%)	23F
5902^15^A	--	27	8	19	15A (68.8%)	9V, 11A, 15BC, 18C
	840	8	3	5	9V (37.5%)	35A (25.0%), 11A, 15BC, 35B
	5336	7	3	4	15A (71.4%)	15BC, 18C
	Other	12	2	10	15A (50.0%), 35A (25.0%)	11A (16.7%), 9V
847^19^A	--	26	18	8	19A (100%)	-
	847	20	13	7	19A	-
	Other	6	5	1	19A	-
499^6^A	--	25	7	18	6A (88.0%)	6A/B, 15BC
	499	21	6	15	6A (95.2%)	15BC
	Other	4	1	3	6A (75.0%), 6B (25.0%)	-
1146^35^B	--	25	5	20	35B (84.0%)	17F, 23F, 29
	1146	16	4	12	35B (87.5%)	23F, 29
	Other	9	1	8	35B (77.8%)	17F
852^10^A	--	19	9	10	10A (89.5%)	10B
	852	18	8	10	10A (88.9%)	10B
	5304	1	1	0	10A	-
914^6^A	--	18	3	15	6A (88.9%)	6B
	5354	13	2	11	6A (84.6%)	6B
	Other	5	1	4	6A	-
989^12^F	--	16	14	2	12F (100%)	-
	989	9	9	0	12F	-
	5352	6	4	2	12F	-
	5797	1	1	0	12F	-

Note: CC = clonal complex; ST = sequence type; ‘-’ indicates that no other serotypes were detected.

a CCs with >15 isolate representatives shown here; STs within a CC that have fewer than 5 isolate representatives are grouped as “Other”.

### Population Genetic Structure Among PCV10 Serotypes

161 serotype 1 isolates from Kilifi were genotyped and all were members of CC217^1^ (note: ST^serotype^; Table 2). Only 3 of the serotype 1 isolates were recovered from carriage, the rest were isolated from patients with invasive disease. It was previously reported that serotype 1 genotypes had a distinctive phylogeography [30]. Ancestral ST217^1^ and its closely related single locus variants (SLVs) were shown to be the predominant serotype 1 STs in Kenya and Israel, and later were also shown to be common in other African countries, namely Ghana, Burkina Faso and The Gambia [31-33]. Other serotype 1 genotypes appear to predominate elsewhere [19,30]. Based on available data CC217^1^ still appears to be largely an African CC [19]. 

Vaccine serotype 4 isolates from Kilifi were predominantly invasive isolates of ST853, which are members of the widely distributed CC246^4^ (Table 2). CC246^4^ has been of recent interest since unique recombinants (a serotype 4 to 19A capsular switch) in the United States emerged from this CC [34-36]. Only 1 isolate of CC246 in Kilifi expressed an alternative serotype and this was serotype 2, a member of a cluster of serotype 2 isolates within CC246 that have also previously been reported in Asia and Africa [19]. 

All of the vaccine serotype 5 isolates (n = 44) were from cases of invasive disease, and all were members of CC289^5^. ST289 is the widely-distributed Pneumococcal Molecular Epidemiology Network (PMEN) clone represented by the Columbia5-19 strain [37] and although it was a common ST (n = 14 isolates), ST245^5^, an SLV of ST289^5^, was more prevalent in Kilifi (n = 26 isolates). 

One major serotype 9V CC was identified (CC706^9V^) and 100% of the CC706 isolates expressed serotype 9V. 39 vaccine serotype 18C isolates were characterised in this study and 35 of these were members of CC1381^18C^. ST1381^18C^ shares only the *xpt* MLST allele with ST113^18C^ (PMEN clone Netherlands^18C^-36), which is a widely-distributed genotype associated with serotype 18C [19]. 

Among vaccine serotypes 6B, 14, 19F and 23F there were two major CCs circulating for each serotype and in each case the two CCs defined a large proportion of the isolate representatives for each serotype: 45% of 107 serotype 6B isolates; 85% of 103 serotype 14 isolates; 64% of 104 serotype 19F isolates; and 76% of 86 serotype 23F isolates (Table 2). One notable observation was that all 24 Kilifi isolates of ST230 expressed serotype 14 and 83% of them were causing invasive disease. CC230 is a widely-distributed CC that is predominantly serotypes 14 and 19A, although ST230 (PMEN clone Denmark14-32) isolates have been reported with alternative serotypes [19]. ST230^19A^ increased in prevalence in the United States (US) and Spain after PCV7 implementation [35,36,38]. In Kilifi, a double locus variant (DLV) of ST230 – ST 700 – was also prevalent, isolates of which exclusively expressed serotype 3. ST700^3^ was the predominant genotype associated with serotype 3 in this Kilifi collection and ST700^3^ isolates have been found elsewhere in Africa. The common globally-distributed serotype 3 genotype is ST180^3^ (PMEN clone Netherlands3-31), and it is only distantly related to ST700^3^ (MLST alleles *gki* and *spi* are shared) [19]. 

### Population Genetic Structure Among Non-PCV10 Serotypes

CCs 701^13,15BC^, 499^6^A, 1146^35^B, 852^10^A, 914^6^A and 5902^15^A were major clones found in Kilifi, predominantly comprised of isolates from carriage rather than invasive disease (Table 3). CC847^19A^ (n = 26 isolates; 100% serotype 19A) is only distantly related (STs share ≤2 MLST alleles) to any of the other major serotype 19A genotypes (e.g. CCs 199, 320/271/236, 695) that circulate in many countries, which have significantly increased post-PCV introduction in the US and elsewhere [35,36,39,40]. 

All serotype 12F isolates (n = 16) from Kilifi were members of CC989^12F^ and isolates of this CC have also been found elsewhere in Africa [19]. The predicted ancestral ST, ST989^12F^, shares only the *spi* MLST allele with the widely-distributed ST218^12F^ (PMEN clone Denmark^12F^-34). Serotype 12F is generally uncommon among carriage isolates, which is also true of this Kilifi dataset (14 of 16 serotype 12F isolates were invasive), and has historically been shown to cause outbreaks [41,42], but it is not included in any currently available PCV. 

### Temporal Variation of CCs Containing Invasive Isolates Collected From 1994-2008.

**Figure 1:**
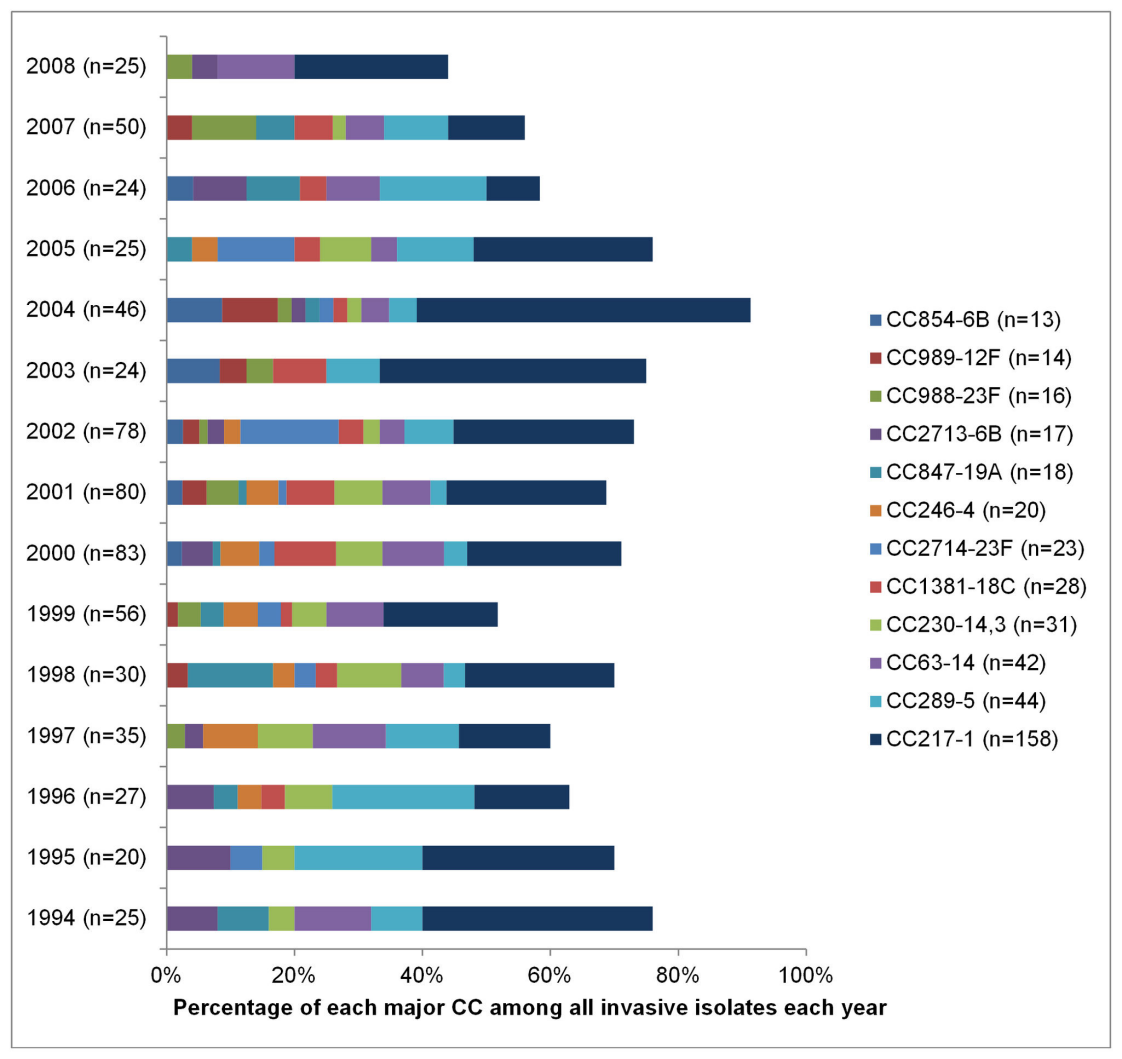
Prevalence of major clonal complexes (CCs) associated with invasive isolates collected in Kilifi. The total number of isolates genotyped each year from 1994-2008 is stated in parentheses in the y-axis labels.

Since the invasive isolate dataset in Kilifi spanned 15 years, we were able to explore temporal variation among circulating STs and CCs over that extended period of time. 12 CCs were comprised of >10 invasive isolate representatives in total over the time period, which represented 68% (424/628) of the entire invasive collection (Figure 1). The prevalence of every CC varied over time, to a greater or lesser extent. Most obvious was the dominance of CC217^1^ (n = 158), which ranged in prevalence from 8 - 52% of all invasive isolates characterised each year. The next most prevalent CC was CC289^5^ (n = 44), which fluctuated in prevalence from 0 - 22% over the period of surveillance. Other major CCs also varied in prevalence over the surveillance years. These longitudinal data will be particularly important in analyses that monitor changes in the prevalence of individual CCs, particularly CCs of nonvaccine serotypes such as CC230 (ST700^3^), CC989^12F^ and CC847^19A^, which ranged from 0-4%, 0-9% and 0-13% of all invasive isolates characterised each year, respectively, prior to PCV10 introduction. Note that CCs not depicted in Figure 1 made only minor contributions to the overall pre-PCV population structure and contributed 0-3 isolates in any given year (data not shown), but these data will be re-evaluated in future post-PCV10 analyses.

**Figure 2:**
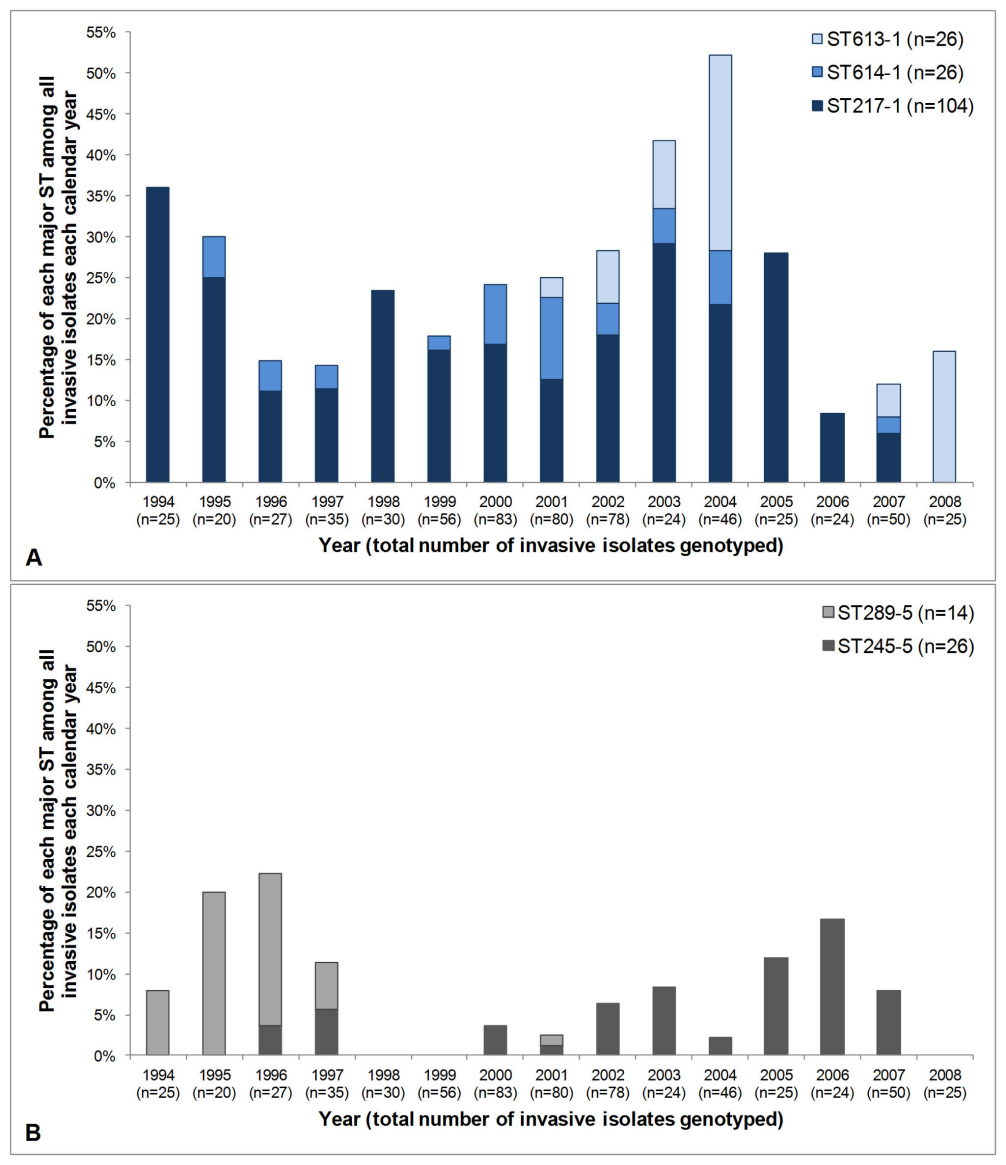
Fluctuation in the prevalence of major sequence types (STs) associated with invasive isolates from Kilifi. Invasive isolates were collected from 1994-2008 and the major STs associated with serotypes 1 (Figure 2A) and 5 (Figure 2B) are depicted in each panel. The total number of isolates genotyped each year (see Methods) is stated in parentheses in the x-axis labels.

**Figure 3:**
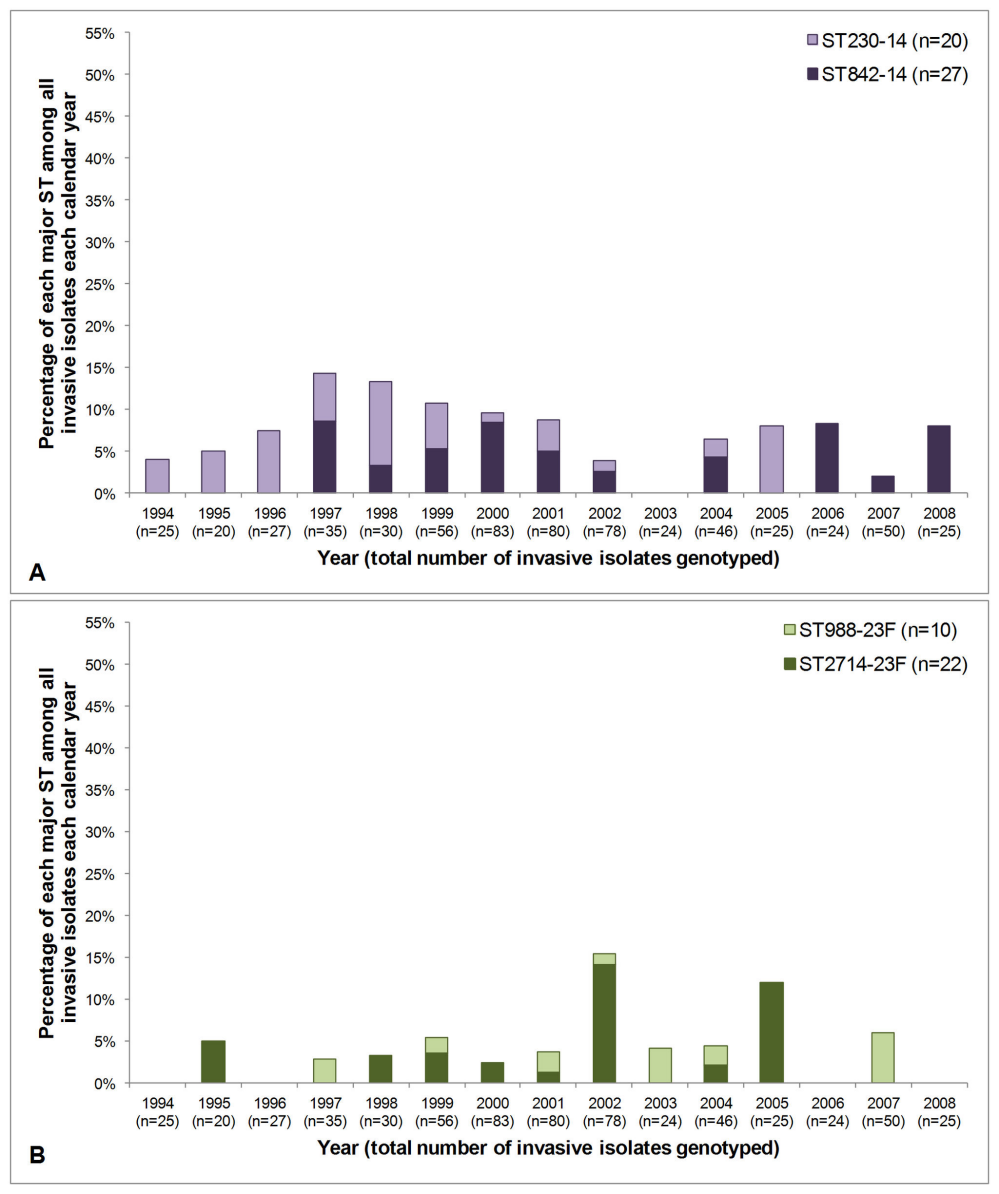
Fluctuation in the prevalence of major sequence types (STs) associated with invasive isolates from Kilifi. Invasive isolates were collected from 1994-2008 and the major STs associated with serotypes 14 (Figure 3A) and 23F (Figure 3B) are depicted in each panel. The total number of isolates genotyped each year (see Methods) is stated in parentheses in the x-axis labels.

Figures 2 and 3 depict temporal variation in the prevalence of several of the major STs circulating among children with invasive disease in Kilifi over the 15 year surveillance period. These STs were selected for analysis because there were multiple major STs (≥10 isolates) that expressed the same serotype. Serotype 1 isolates were recovered every year of surveillance and the three major STs associated with serotype 1 are all closely related: ST217 is the predicted ancestor and ST613 and ST614 are SLVs of ST217. Either two or three of these STs were represented in 10 of the 15 surveillance years, and in the remaining five years only one ST was detected (Figure 2A). Figure 2B depicts the two major serotype 5 STs (which are SLVs of each other), but despite their genetic similarity at an ST level these STs were more restricted with respect to when they were detected, i.e. ST289^5^ was predominant in the early years of surveillance, but was replaced by ST245^5^ in the latter years. In contrast, Figures 3A and 3B depict the prevalence of pairs of unrelated serotype 14 and 23F genotypes, respectively, in each calendar year. The two serotype 14 STs were circulating concomitantly in half of the surveillance years, and both serotype 23F STs were detected in four of the surveillance years (note that only 10 isolates of ST988^23F^ were detected overall). 

**Figure 4:**
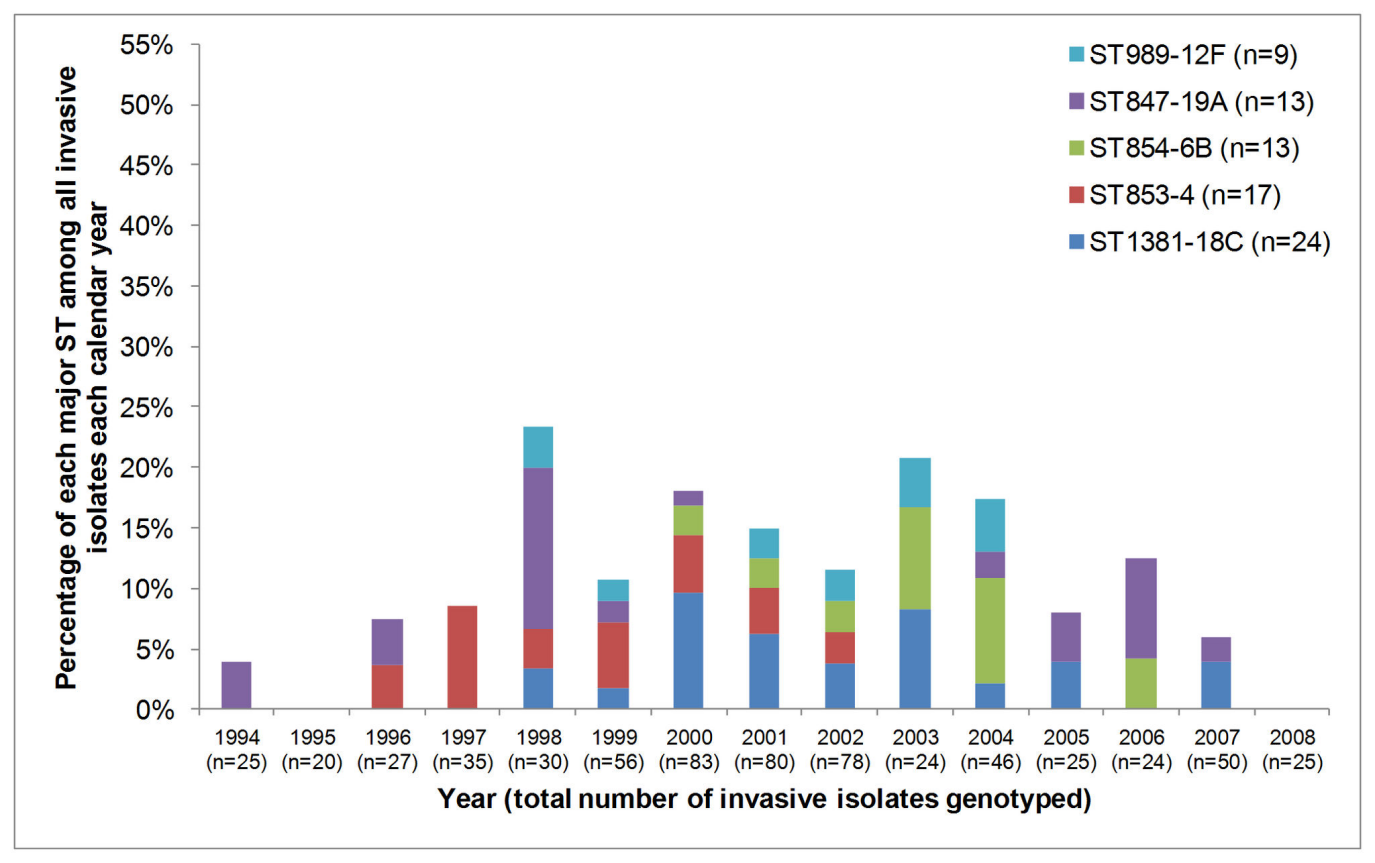
Five additional major sequence types (STs) associated with invasive isolates that circulated in Kilifi. Major STs associated with serotypes 12F, 19A, 6B, 4 and 18C are depicted for each surveillance year from 1994-2008. The total number of isolates genotyped each year (see Methods) is stated in parentheses in the x-axis labels.

Figure 4 completes the picture for the circulating major STs, demonstrating that although only one major genotype predominated for each of these serotypes, none of these major genotypes circulated every year throughout the surveillance period. Although a frequency of ≥10 isolates was used as the criterion for “major” ST and only nine isolates of ST989^12F^ were recovered over the study period, the ST989^12F^ data are shown to demonstrate that there was no serotype 12F epidemic over this surveillance period.

## Discussion

The introduction of PCV10 is vitally important for Kenya since the morbidity and mortality associated with pneumococcal disease is high. The incidence of invasive disease associated with PCV10 serotypes has decreased since vaccination began [43], with a marked attenuation of morbidity and mortality among vaccinated children, and potentially also among unvaccinated older children and adults if herd protection proves to be sufficient. It is expected that introduction of PCV10 in Kenya will perturb the pneumococcal population genetic structure, but a key question is whether or not these perturbations will diminish the overall benefit of vaccine introduction by resulting in increased nonvaccine serotype disease. By comparison, the US has used PCV for the longest period of time and the most notable nonvaccine serotype increase has been that of serotype 19A disease, which was in part due to the emergence of a novel genotype [34-36]. Importantly, this new recombinant (ST695^19A^) quickly became the third most common serotype 19A CC causing invasive disease in the US [35], which emphasises the importance of detecting such population-based genetic changes. 

Post-vaccine changes in the US were revealed because the pre-vaccine pneumococcal population genetic structure in the US was well defined. Active, population- and laboratory-based invasive disease surveillance has been on-going in the US since 1995 (5 years before PCV was introduced) and two studies specifically characterised large, representative collections of pneumococci to identify the pre-PCV7 baseline set of genotypes circulating in the US [14,20]. The Kilifi pneumococcal invasive disease surveillance programme is also active, population- and laboratory-based, and collected isolates and data for 15 years prior to PCV introduction. Several carefully designed pneumococcal carriage studies have also been performed during that time [8,25]. The data (patient demographics, clinical outcome, serotype) combined with the genotyping work described here provide a comprehensive description of the pre-vaccine pneumococcal population in Kilifi.

MLST has been used to characterise many thousands of pneumococci collected across Europe, North and South America, Australia and Asia and many of the major STs within countries have disseminated across continents, e.g. STs 81^23^F^,19F^, 90^6^B, 156^9^V, 9^14^, 124^14^, 113^18^C, 218^12^F, 191^7^F, 180^3^, 199^19^A [19]. Many such STs have been identified as PMEN clones, in part defined by their widespread nature [19,37]. Our study demonstrated that although the major serotypes found in Kilifi and Africa are similar to those found elsewhere in the world, most of the major STs/CCs are different, at least as far as we can tell from the data in the MLST database. It is important to note that the contribution of data to the MLST database is voluntary – investigators must submit new alleles and new STs to the database for assignment, but only rarely do investigators submit their entire study dataset. Once an ST is added to the database, there is no obligation for another investigator who subsequently detects that ST elsewhere to indicate this to the curators. Thus, the MLST database reliably captures allelic diversity, but we do not have all the information about all isolates genotyped to know for certain whether some of the STs detected in Kilifi are truly African in origin, even though that is what the database suggests. However, with those caveats, the MLST database currently contains over 9,000 STs and more than 21,000 isolates recovered from all over the globe, and we do know that Africa is generally under-represented in the MLST database as compared to other geographical regions. Therefore these data significantly increase our understanding of the pneumococcal population structure in Kilifi, and possibly in Africa more broadly. However, since many of the major STs identified in Kilifi were different to the major STs of the same serotypes that circulate globally, we have little additional knowledge on which to predict their likely increase or evolution and therefore follow-up studies will be essential. 

An interesting observation was the temporal fluctuation in STs that expressed the same serotypes, accepting that for some of these serotypes the numbers observed each year were small. There were no major changes in Kilifi District (e.g. changes in laboratory methods or practice, antimicrobial use or stewardship, early vaccine uptake, etc.) over the surveillance period that would have contributed to big fluctuations in the circulating serotypes or genotypes. The prevalence of circulating serotypes is known to vary and thus temporal fluctuations in circulating serotypes and genotypes in Kilifi were not surprising [44-47]; however, our data might suggest that for serotypes 1, 14 and 23F it mattered less which of the different STs associated with these serotypes was circulating. Previously published papers have debated whether it is the serotype or MLST genotype that plays a more important role in the potential for an isolate to cause invasive disease [22,48-50]. The argument favouring a primary role of serotype is supported by the fact that in Kilifi different major genotypes expressing the same serotype were detected concomitantly. All three serotype 1 STs were closely related, but the pairs of serotype 14 and 23F STs were unrelated. Alternatively, the two serotype 5 STs, although closely related at the MLST loci, appeared to be more restricted in their circulation. Perhaps the two serotype 5 STs differ markedly elsewhere in the genome, and as a result one serotype 5 genotype can outcompete the other and make co-circulation less likely. It is known that the immunological response differs for each pneumococcal serotype [51], and thus it may be the serotype-specific immune response that largely determines the circulation of serotypes regardless of which genotypic backbone they maintain. More likely, it is a particular combination of serotype (and corresponding immunity within the human population) and genotype that is the best explanation for the temporal patterns of ST circulation we observed in Kilifi. 

Several major CCs were comprised of nonvaccine serotype isolates (10A, 13, 15A, 15BC and 35B) primarily recovered from healthy children, although they were also recovered from children with invasive disease so the potential for serotype replacement disease remains. Serotypes 10A, 15BC and 35B increased in prevalence in the US post-PCV7 vaccine [14,15,23,52], but the predominant STs were different to those found in Kilifi so the predictive power based on genotype is minimal [14,23,24]. Nonvaccine serotypes 3, 12F and 19A were already prevalent prior to vaccine introduction and their ability to cause invasive disease in Kilifi and elsewhere is clear. Serotypes 3 and 19A significantly increased in prevalence post-vaccine implementation in the US, although serotype 12F significantly decreased [14,15]. Serotype 19A has also significantly increased elsewhere [40,53]. 

In this study we established the baseline set of genotypes in Kilifi prior to PCV10 introduction, which will allow for the detection of changes in prevalence of pre-existing STs and the identification of new nonvaccine STs (as putative imports or new recombinants). It will be essential that any perceived increases or decreases in serotype or genotype prevalence after PCV10 vaccination is established in Kilifi be considered in the context of the pre-vaccine genotypic landscape.

## Supporting Information

Table S1 **Clonal complexes and sequence types found in the invasive pneumococcal collection, stratified by serotype.**
(DOCX)

Table S2 **Clonal complexes and sequence types found in the carriage pneumococcal collection, stratified by serotype.**
(DOCX)
