# Supplementary material for: Population Genetic Structure of Streptococcus pneumoniae in Kilifi, Kenya, Prior to the Introduction of Pneumococcal Conjugate Vaccine
Source: PLoS One. 2013 Nov 25;8(11):e81539. doi: 10.1371/journal.pone.0081539 (PMC3839905; doi:10.1371/journal.pone.0081539)
Supplement: Table S1 — Clonal complexes and sequence types found in the invasive pneumococcal collection, stratified by serotype. (DOCX) [file pone.0081539.s001.docx]

| **Table S1. Clonal complexes and sequence types found in the invasive pneumococcal collection, stratified by serotype.** | | | | | |
| --- | --- | --- | --- | --- | --- |
| **Serotype** | **No. of isolates** | **Clonal complex** | **Sequence type** | **No. of isolates** | |
| **1** | **158** | **-** | **-** | **-** | |
|  |  | **CC217** | **-** | **158** | |
|  |  |  | 217 | 104 | |
|  |  |  | 613 | 26 | |
|  |  |  | 614 | 26 | |
|  |  |  | 5916 | 1 | |
|  |  |  | 5919 | 1 | |
| **2** | **1** | **-** | **-** | **-** | |
|  |  | **CC246** | **-** | **1** | |
|  |  |  | 74 | 1 | |
| **3** | **15** | **-** | **-** | **-** | |
|  |  | **CC230** | **-** | **9** | |
|  |  |  | 700 | 8 | |
|  |  |  | 6079 | 1 | |
|  |  | **CC5889/5900** | **-** | **3** | |
|  |  |  | 5889 | 1 | |
|  |  |  | 5900 | 2 | |
|  |  | **CC458** | **-** | **1** | |
|  |  |  | 458 | 1 | |
|  |  | **CC848** | **-** | **1** | |
|  |  |  | 848 | 1 | |
|  |  | **CC5895/6084** | **-** | **1** | |
|  |  |  | 5895 | 1 | |
| **4** | **23** | **-** | **-** | **-** | |
|  |  | **CC246** | **-** | **19** | |
|  |  |  | 853 | 17 | |
|  |  |  | 6082 | 1 | |
|  |  |  | 6091 | 1 | |
|  |  | **Singleton** | **-** | **3** | |
|  |  |  | 5913 | 1 | |
|  |  |  | 6073 | 1 | |
|  |  |  | 6087 | 1 | |
|  |  | **CC2212/5097/6089** | **-** | **1** | |
|  |  |  | 6089 | 1 | |
| **5** | **45** | **-** | **-** | **-** | |
|  |  | **CC289** | **-** | **44** | |
|  |  |  | 245 | 26 | |
|  |  |  | 289 | 14 | |
|  |  |  | 5896 | 2 | |
|  |  |  | 5915 | 1 | |
|  |  |  | 6075 | 1 | |
|  |  | **CC3035/4840** | **-** | **1** | |
|  |  |  | 4840 | 1 | |
| **6A** | **41** | **-** | **-** | **-** |  |
|  |  | **Singleton** | **-** | **13** |  |
|  |  |  | 845 | 4 |  |
|  |  |  | 5251 | 1 |  |
|  |  |  | 5318 | 1 |  |
|  |  |  | 5321 | 4 |  |
|  |  |  | 5376 | 1 |  |
|  |  |  | 5880 | 1 |  |
|  |  |  | 6071 | 1 |  |
|  |  | **CC499** | **-** | **7** |  |
|  |  |  | 499 | 6 |  |
|  |  |  | 5907 | 1 |  |
|  |  | **CC7063** | **-** | **6** |  |
|  |  |  | 5327 | 3 |  |
|  |  |  | 5885 | 1 |  |
|  |  |  | 6072 | 1 |  |
|  |  |  | 6078 | 1 |  |
|  |  | **CC5329/5876** | **-** | **5** |  |
|  |  |  | 5325 | 1 |  |
|  |  |  | 5329 | 3 |  |
|  |  |  | 5894 | 1 |  |
|  |  | **CC854** | **-** | **3** |  |
|  |  |  | 854 | 3 |  |
|  |  | **CC5879** | **-** | **3** |  |
|  |  |  | 5879 | 1 |  |
|  |  |  | 6080 | 1 |  |
|  |  |  | 6086 | 1 |  |
|  |  | **CC473** | **-** | **2** |  |
|  |  |  | 5256 | 2 |  |
|  |  | **CC914** | **-** | **2** |  |
|  |  |  | 914 | 1 |  |
|  |  |  | 5354 | 1 |  |
| **6B** | **55** | **-** | **-** | **-** |  |
|  |  | **CC2713** | **-** | **17** |  |
|  |  |  | 2713 | 7 |  |
|  |  |  | 5301 | 1 |  |
|  |  |  | 5302 | 6 |  |
|  |  |  | 5789 | 1 |  |
|  |  |  | 5904 | 1 |  |
|  |  |  | 6097 | 1 |  |
|  |  | **CC854** | **-** | **10** |  |
|  |  |  | 854 | 10 |  |
|  |  | **CC990** | **-** | **7** |  |
|  |  |  | 990 | 7 |  |
|  |  | **Singleton** | **-** | **6** |  |
|  |  |  | 4217 | 1 |  |
|  |  |  | 5322 | 1 |  |
|  |  |  | 5376 | 1 |  |
|  |  |  | 5883 | 1 |  |
|  |  |  | 5914 | 1 |  |
|  |  |  | 6083 | 1 |  |
|  |  | **CC176** | **-** | **5** |  |
|  |  |  | 172 | 5 |  |
|  |  | **CC6085/6115** | **-** | **2** |  |
|  |  |  | 6085 | 2 |  |
|  |  | **CC5879** | **-** | **2** |  |
|  |  |  | 5798 | 1 |  |
|  |  |  | 5910 | 1 |  |
|  |  | **CC914** | **-** | **1** |  |
|  |  |  | 5354 | 1 |  |
|  |  | **CC146** | **-** | **1** |  |
|  |  |  | 5801 | 1 |  |
|  |  | **CC5878** | **-** | **1** |  |
|  |  |  | 5877 | 1 |  |
|  |  | **CC2779** | **-** | **1** |  |
|  |  |  | 5271 | 1 |  |
|  |  | **CC5895/6084** | **-** | **1** |  |
|  |  |  | 6084 | 1 |  |
|  |  | **CC4368** | **-** | **1** |  |
|  |  |  | 5920 | 1 |  |
| **7C** | **1** | **-** | **-** | **-** |  |
|  |  | **Singleton** | **-** | **1** |  |
|  |  |  | 8864 | 1 |  |
| **7F** | **3** | **-** | **-** | **-** |  |
|  |  | **CC28/5067** | **-** | **3** |  |
|  |  |  | 5330 | 1 |  |
|  |  |  | 5892 | 1 |  |
|  |  |  | 6076 | 1 |  |
| **9A** | **1** | **-** | **-** | **-** |  |
|  |  | **CC4881** | **-** | **1** |  |
|  |  |  | 5778 | 1 |  |
| **9L** | **2** | **-** | **-** | **-** |  |
|  |  | **Singleton** | **-** | **1** |  |
|  |  |  | 6106 | 1 |  |
|  |  | **CC4959/5315** | **-** | **1** |  |
|  |  |  | 5315 | 1 |  |
| **9V** | **15** | **-** | **-** | **-** |  |
|  |  | **CC706** | **-** | **5** |  |
|  |  |  | 706 | 2 |  |
|  |  |  | 5263 | 1 |  |
|  |  |  | 5283 | 2 |  |
|  |  | **CC5902** | **-** | **4** |  |
|  |  |  | 840 | 3 |  |
|  |  |  | 5902 | 1 |  |
|  |  | **Singleton** | **-** | **2** |  |
|  |  |  | 5324 | 2 |  |
|  |  | **CC4881** | **-** | **2** |  |
|  |  |  | 4908 | 1 |  |
|  |  |  | 5897 | 1 |  |
|  |  | **CC701** | **-** | **2** |  |
|  |  |  | 701 | 2 |  |
| **10A** | **10** | **-** | **-** | **-** |  |
|  |  | **CC852** |  | **9** |  |
|  |  |  | 852 | 8 |  |
|  |  |  | 5304 | 1 |  |
|  |  | **CC3735/4084/5521** |  | **1** |  |
|  |  |  | 5781 | 1 |  |
| **10F** | **2** | **-** | **-** | **-** |  |
|  |  | **Singleton** |  | **2** |  |
|  |  |  | 5261 | 2 |  |
| **11A** | **3** | **-** | **-** | **-** |  |
|  |  | **CC5752** |  | **3** |  |
|  |  |  | 5752 | 1 |  |
|  |  |  | 5884 | 2 |  |
| **12F** | **14** | **-** | **-** | **-** |  |
|  |  | **CC989** | **-** | **14** |  |
|  |  |  | 989 | 9 |  |
|  |  |  | 5352 | 4 |  |
|  |  |  | 5797 | 1 |  |
| **13** | **2** | **-** | **-** | **-** |  |
|  |  | **CC701** |  | **2** |  |
|  |  |  | 701 | 2 |  |
| **14** | **77** | **-** | **-** | **-** |  |
|  |  | **CC63** | **-** | **42** |  |
|  |  |  | 63 | 1 |  |
|  |  |  | 842 | 27 |  |
|  |  |  | 2716 | 5 |  |
|  |  |  | 5187 | 1 |  |
|  |  |  | 5257 | 1 |  |
|  |  |  | 5259 | 4 |  |
|  |  |  | 5793 | 1 |  |
|  |  |  | 5908 | 1 |  |
|  |  |  | 5917 | 1 |  |
|  |  | **CC230** | **-** | **22** |  |
|  |  |  | 230 | 20 |  |
|  |  |  | 5320 | 1 |  |
|  |  |  | 5912 | 1 |  |
|  |  | **Singleton** | **-** | **7** |  |
|  |  |  | 850 | 1 |  |
|  |  |  | 5786 | 1 |  |
|  |  |  | 5886 | 1 |  |
|  |  |  | 5898 | 1 |  |
|  |  |  | 6069 | 1 |  |
|  |  |  | 6077 | 1 |  |
|  |  |  | 6094 | 1 |  |
|  |  | **CC844** | **-** | **1** |  |
|  |  |  | 844 | 1 |  |
|  |  | **CC5368/7053** | **-** | **1** |  |
|  |  |  | 5368 | 1 |  |
|  |  | **CC5879** | **-** | **1** |  |
|  |  |  | 5798 | 1 |  |
|  |  | **CC988** | **-** | **1** |  |
|  |  |  | 5253 | 1 |  |
|  |  | **CC2715** | **-** | **1** |  |
|  |  |  | 6088 | 1 |  |
|  |  | **CC703** | **-** | **1** |  |
|  |  |  | 703 | 1 |  |
| **15A** | **5** | **-** | **-** | **-** |  |
|  |  | **CC5902** | **-** | **3** |  |
|  |  |  | 991 | 1 |  |
|  |  |  | 5336 | 2 |  |
|  |  | **CC703** | **-** | **2** |  |
|  |  |  | 5249 | 1 |  |
|  |  |  | 5277 | 1 |  |
| **15BC** | **3** | **-** | **-** | **-** |  |
|  |  | **CC5484/5796** | **-** | **1** |  |
|  |  |  | 5484 | 1 |  |
|  |  | **CC701** | **-** | **1** |  |
|  |  |  | 701 | 1 |  |
|  |  | **CC4209** | - | **1** |  |
|  |  |  | 5792 | 1 |  |
| **16F** | **3** | **-** | **-** | **-** |  |
|  |  | **CC5250/5947** | **-** | **2** |  |
|  |  |  | 5250 | 2 |  |
|  |  | **Singleton** | - | **1** |  |
|  |  |  | 5326 | 1 |  |
| **18B** | **1** | **-** | **-** | **-** |  |
|  |  | **CC1381** | **-** | **1** |  |
|  |  |  | 6093 | 1 |  |
| **18C** | **31** | **-** | **-** | **-** |  |
|  |  | **CC1381** | **-** | **26** |  |
|  |  |  | 1381 | 24 |  |
|  |  |  | 5284 | 1 |  |
|  |  |  | 5903 | 1 |  |
|  |  | **CC5878** | **-** | **2** |  |
|  |  |  | 5878 | 2 |  |
|  |  | **CC28/5067** | **-** | **1** |  |
|  |  |  | 28 | 1 |  |
|  |  | **Singleton** | **-** | **1** |  |
|  |  |  | 5891 | 1 |  |
|  |  | **CC5902** | **-** | **1** |  |
|  |  |  | 5336 | 1 |  |
| **18F** | **3** | **-** | **-** | **-** |  |
|  |  | **CC5287** | **-** | **2** |  |
|  |  |  | 2711 | 1 |  |
|  |  |  | 5287 | 1 |  |
|  |  | **CC1381** | **-** | **1** |  |
|  |  |  | 5795 | 1 |  |
| **19A** | **20** | **-** | **-** | **-** |  |
|  |  | **CC847** | **-** | **18** |  |
|  |  |  | 847 | 13 |  |
|  |  |  | 4162 | 1 |  |
|  |  |  | 5262 | 1 |  |
|  |  |  | 5270 | 1 |  |
|  |  |  | 5905 | 1 |  |
|  |  |  | 5906 | 1 |  |
|  |  | **Singleton** | **-** | **2** |  |
|  |  |  | 5372 | 1 |  |
|  |  |  | 5791 | 1 |  |
| **19B** | **1** | **-** | **-** | **-** |  |
|  |  | **Singleton** | **-** | **1** |  |
|  |  |  | 5779 | 1 |  |
| **19F** | **24** | **-** | **-** | **-** |  |
|  |  | **CC844** | **-** | **9** |  |
|  |  |  | 844 | 5 |  |
|  |  |  | 5268 | 1 |  |
|  |  |  | 5269 | 1 |  |
|  |  |  | 5339 | 2 |  |
|  |  | **CC2715** | **-** | **8** |  |
|  |  |  | 2715 | 4 |  |
|  |  |  | 6088 | 3 |  |
|  |  |  | 6095 | 1 |  |
|  |  | **CC347** | **-** | **2** |  |
|  |  |  | 556 | 1 |  |
|  |  |  | 6074 | 1 |  |
|  |  | **Singleton** | **-** | **1** |  |
|  |  |  | 5319 | 1 |  |
|  |  | **CC703** | **-** | **1** |  |
|  |  |  | 6070 | 1 |  |
|  |  | **CC5484/5796** | **-** | **1** |  |
|  |  |  | 5796 | 1 |  |
|  |  | **CC2386/5760** | **-** | **1** |  |
|  |  |  | 2386 | 1 |  |
|  |  | **CC3518** | **-** | **1** |  |
|  |  |  | 3518 | 1 |  |
| **21** | **1** | **-** | **-** | **-** |  |
|  |  | **Singleton** | **-** | **1** |  |
|  |  |  | 6092 | 1 |  |
| **22A** | **1** | **-** | **-** | **-** |  |
|  |  | **CC5266** | **-** | **1** |  |
|  |  |  | 5266 | 1 |  |
| **23B** | **2** | **-** | **-** | **-** |  |
|  |  | **CC5370** | **-** | **2** |  |
|  |  |  | 5370 | 2 |  |
| **23F** | **44** | **-** | **-** | **-** |  |
|  |  | **CC2714** | **-** | **23** |  |
|  |  |  | 2714 | 22 |  |
|  |  |  | 5799 | 1 |  |
|  |  | **CC988** | **-** | **14** |  |
|  |  |  | 988 | 10 |  |
|  |  |  | 5253 | 1 |  |
|  |  |  | 5780 | 1 |  |
|  |  |  | 5909 | 1 |  |
|  |  |  | 5911 | 1 |  |
|  |  | **Singleton** | **-** | **4** |  |
|  |  |  | 5311 | 1 |  |
|  |  |  | 5321 | 1 |  |
|  |  |  | 5882 | 1 |  |
|  |  |  | 6081 | 1 |  |
|  |  | **CC848** | **-** | **2** |  |
|  |  |  | 848 | 2 |  |
|  |  | **CC1146** | **-** | **1** |  |
|  |  |  | 1146 | 1 |  |
| **24F** | **4** | **-** | **-** | **-** |  |
|  |  | **CC4888/5077** | **-** | **2** |  |
|  |  |  | 5077 | 2 |  |
|  |  | **CC2208** | **-** | **2** |  |
|  |  |  | 2818 | 2 |  |
| **28F** | **1** | **-** | **-** | **-** |  |
|  |  | **Singleton** | **-** | **1** |  |
|  |  |  | 5881 | 1 |  |
| **29** | **4** | **-** | **-** | **-** |  |
|  |  | **CC5329/5876** | **-** | **2** |  |
|  |  |  | 5875 | 1 |  |
|  |  |  | 5876 | 1 |  |
|  |  | **CC1146** | **-** | **2** |  |
|  |  |  | 1146 | 1 |  |
|  |  |  | 5788 | 1 |  |
| **33B** | **1** | **-** | **-** | **-** |  |
|  |  | **CC5333/5901** | **-** | **1** |  |
|  |  |  | 5901 | 1 |  |
| **34** | **3** | **-** | **-** | **-** |  |
|  |  | **CC841/5258** |  | **3** |  |
|  |  |  | 5258 | 3 |  |
| **35B** | **5** | **-** | **-** | **-** |  |
|  |  | **CC1146** | **-** | **2** |  |
|  |  |  | 1146 | 2 |  |
|  |  | **CC176** | **-** | **2** |  |
|  |  |  | 172 | 2 |  |
|  |  | **CC988** | **-** | **1** |  |
|  |  |  | 5921 | 1 |  |
| **38** | **2** | **-** | **-** | **-** |  |
|  |  | **CC5560/6090/6103** |  |  |  |
|  |  |  | 6090 | 1 |  |
|  |  | **CC393** |  | **1** |  |
|  |  |  | 5918 | 1 |  |
| **Total** | **627** | **-** | **-** | **-** |  |
