# Supplementary material for: Population Genetic Structure of Streptococcus pneumoniae in Kilifi, Kenya, Prior to the Introduction of Pneumococcal Conjugate Vaccine
Source: PLoS One. 2013 Nov 25;8(11):e81539. doi: 10.1371/journal.pone.0081539 (PMC3839905; doi:10.1371/journal.pone.0081539)
Supplement: Table S2 — Clonal complexes and sequence types found in the carriage pneumococcal collection, stratified by serotype. (DOCX) [file pone.0081539.s002.docx]

| **Table S2. Clonal complexes and sequence types found in the carriage pneumococcal collection, stratified by serotype.** | | | | |
| --- | --- | --- | --- | --- |
|  |  |  |  |  |
| **Serotype** | **No. of isolates** | **Clonal complex** | **Sequence type** | **No. of isolates** |
| **1** | **3** | **-** | **-** | **-** |
|  |  | **CC217** | **-** | **3** |
|  |  |  | 217 | 2 |
|  |  |  | 613 | 1 |
| **3** | **5** | **-** | **-** | **-** |
|  |  | **CC230** | **-** | **4** |
|  |  |  | 700 | 3 |
|  |  |  | 5937 | 1 |
|  |  | **CC458** | **-** | **1** |
|  |  |  | 458 | 1 |
| **4** | **6** | **-** | **-** | **-** |
|  |  | **CC246** | **-** | **4** |
|  |  |  | 853 | 3 |
|  |  |  | 5757 | 1 |
|  |  | **CC988** | **-** | **1** |
|  |  |  | 5342 | 1 |
|  |  | **CC2213** | **-** | **1** |
|  |  |  | 4437 | 1 |
| **6A** | **52** | **-** | **-** | **-** |
|  |  | **CC499** | **-** | **15** |
|  |  |  | 499 | 13 |
|  |  |  | 5761 | 1 |
|  |  |  | 5941 | 1 |
|  |  | **CC914** | **-** | **14** |
|  |  |  | 5354 | 10 |
|  |  |  | 5362 | 1 |
|  |  |  | 5386 | 3 |
|  |  | **Singleton** | **-** | **7** |
|  |  |  | 5321 | 4 |
|  |  |  | 5335 | 1 |
|  |  |  | 5928 | 1 |
|  |  |  | 5936 | 1 |
|  |  | **CC5294/6335** | **-** | **5** |
|  |  |  | 5294 | 5 |
|  |  | **CC473** | **-** | **3** |
|  |  |  | 2285 | 1 |
|  |  |  | 5334 | 1 |
|  |  |  | 5381 | 1 |
|  |  | **CC5329/5876** | **-** | **3** |
|  |  |  | 5329 | 1 |
|  |  |  | 5767 | 1 |
|  |  |  | 6098 | 1 |
|  |  | **CC2713** | **-** | **1** |
|  |  |  | 6097 | 1 |
|  |  | **CC854** | **-** | **1** |
|  |  |  | 854 | 1 |
|  |  | **CC5752** | **-** | **1** |
|  |  |  | 5752 | 1 |
|  |  | **CC7063** | **-** | **1** |
|  |  |  | 5946 | 1 |
|  |  | **CC5375/5938** | **-** | **1** |
|  |  |  | 5938 | 1 |
| **6B** | **51** | **-** | **-** | **-** |
|  |  | **CC854** | **-** | **16** |
|  |  |  | 854 | 14 |
|  |  |  | 5770 | 1 |
|  |  |  | 5774 | 1 |
|  |  | **CC990** | **-** | **7** |
|  |  |  | 990 | 3 |
|  |  |  | 5380 | 1 |
|  |  |  | 5384 | 1 |
|  |  |  | 5397 | 2 |
|  |  | **CC2713** | **-** | **5** |
|  |  |  | 2713 | 3 |
|  |  |  | 5379 | 2 |
|  |  | **Singleton** | **-** | **4** |
|  |  |  | 5395 | 1 |
|  |  |  | 5775 | 1 |
|  |  |  | 6107 | 1 |
|  |  |  | 6108 | 1 |
|  |  | **CC5879** | **-** | **3** |
|  |  |  | 5798 | 3 |
|  |  | **CC701** | **-** | **3** |
|  |  |  | 701 | 3 |
|  |  | **CC2779** | **-** | **2** |
|  |  |  | 2779 | 1 |
|  |  |  | 5765 | 1 |
|  |  | **CC4368** | **-** | **2** |
|  |  |  | 4368 | 1 |
|  |  |  | 5949 | 1 |
|  |  | **CC499** | **-** | **2** |
|  |  |  | 499 | 1 |
|  |  |  | 5332 | 1 |
|  |  | **CC5329/5876** | **-** | **2** |
|  |  |  | 5378 | 1 |
|  |  |  | 5927 | 1 |
|  |  | **CC6085/6115** | **-** | **1** |
|  |  |  | 6115 | 1 |
|  |  | **CC914** | **-** | **1** |
|  |  |  | 5354 | 1 |
|  |  | **CC176** | **-** | **1** |
|  |  |  | 385 | 1 |
|  |  | **CC473** | **-** | **1** |
|  |  |  | 5334 | 1 |
|  |  | **CC5375/5938** | **-** | **1** |
|  |  |  | 5375 | 1 |
| **7C** | **8** | **-** | **-** | **-** |
|  |  | **CC846/2054/5346** | **-** | **6** |
|  |  |  | 2054 | 5 |
|  |  |  | 5346 | 1 |
|  |  | **Singleton** | **-** | **1** |
|  |  |  | 5348 | 1 |
|  |  | **CC5368/7053** | **-** | **1** |
|  |  |  | 5338 | 1 |
| **8** | **2** | **-** | **-** | **-** |
|  |  | **CC2234/6051** | **-** | **2** |
|  |  |  | 2234 | 2 |
| **9L** | **1** | **-** | **-** | **-** |
|  |  | **Singleton** | **-** | **1** |
|  |  |  | 6106 | 1 |
| **9N** | **1** | **-** | **-** | **-** |
|  |  | **CC3983/6485** | **-** | **1** |
|  |  |  | 3983 | 1 |
| **9V** | **15** | **-** | **-** | **-** |
|  |  | **CC706** | **-** | **12** |
|  |  |  | 706 | 5 |
|  |  |  | 5283 | 4 |
|  |  |  | 5390 | 1 |
|  |  |  | 5764 | 1 |
|  |  |  | 6114 | 1 |
|  |  | **Singleton** | **-** | **1** |
|  |  |  | 5361 | 1 |
|  |  | **CC1871** | **-** | **1** |
|  |  |  | 5363 | 1 |
|  |  | **CC4881** | **-** | **1** |
|  |  |  | 5758 | 1 |
| **10A** | **10** | **-** | **-** | **-** |
|  |  | **CC852** | **-** | **8** |
|  |  |  | 852 | 8 |
|  |  | **Singleton** | **-** | **1** |
|  |  |  | 5755 | 1 |
|  |  | **CC5329/5876** | **-** | **1** |
|  |  |  | 5329 | 1 |
| **10B** | **3** | **-** | **-** | **-** |
|  |  | **CC852** | **-** | **2** |
|  |  |  | 852 | 2 |
|  |  | **Singleton** | **-** | **1** |
|  |  |  | 5944 | 1 |
| **10F** | **1** | **-** | **-** | **-** |
|  |  | **CC909** | **-** | **1** |
|  |  |  | 909 | 1 |
| **11A** | **17** | **-** | **-** | **-** |
|  |  | **CC5752** | **-** | **8** |
|  |  |  | 843 | 2 |
|  |  |  | 5356 | 1 |
|  |  |  | 5752 | 2 |
|  |  |  | 5768 | 1 |
|  |  |  | 5933 | 1 |
|  |  |  | 5940 | 1 |
|  |  | **Singleton** | **-** | **4** |
|  |  |  | 5345 | 4 |
|  |  | **CC5902** | **-** | **3** |
|  |  |  | 840 | 1 |
|  |  |  | 5364 | 1 |
|  |  |  | 5399 | 1 |
|  |  | **CC844** | **-** | **1** |
|  |  |  | 5339 | 1 |
|  |  | **CC6025** | **-** | **1** |
|  |  |  | 5393 | 1 |
| **12F** | **2** | **-** | **-** | **-** |
|  |  | **CC989** | **-** | **2** |
|  |  |  | 5352 | 2 |
| **13** | **13** | **-** | **-** | **-** |
|  |  | **CC701** | **-** | **13** |
|  |  |  | 701 | 9 |
|  |  |  | 1144 | 1 |
|  |  |  | 2053 | 1 |
|  |  |  | 5337 | 1 |
|  |  |  | 6110 | 1 |
| **14** | **27** | **-** | **-** | **-** |
|  |  | **CC63** | **-** | **19** |
|  |  |  | 842 | 17 |
|  |  |  | 6105 | 2 |
|  |  | **CC230** | **-** | **5** |
|  |  |  | 230 | 4 |
|  |  |  | 5369 | 1 |
|  |  | **Singleton** | **-** | **2** |
|  |  |  | 5383 | 1 |
|  |  |  | 5948 | 1 |
|  |  | **CC1264** | **-** | **1** |
|  |  |  | 1264 | 1 |
| **15A** | **11** | **-** | **-** | **-** |
|  |  | **CC5902** | **-** | **8** |
|  |  |  | 991 | 3 |
|  |  |  | 2318 | 1 |
|  |  |  | 5336 | 3 |
|  |  |  | 5924 | 1 |
|  |  | **CC703** | **-** | **3** |
|  |  |  | 703 | 2 |
|  |  |  | 5347 | 1 |
| **15BC** | **20** | **-** | **-** | **-** |
|  |  | **CC701** | **-** | **9** |
|  |  |  | 701 | 1 |
|  |  |  | 5340 | 6 |
|  |  |  | 5922 | 1 |
|  |  |  | 6109 | 1 |
|  |  | **CC1264** | **-** | **2** |
|  |  |  | 1264 | 1 |
|  |  |  | 5389 | 1 |
|  |  | **Singleton** | **-** | **4** |
|  |  |  | 5374 | 2 |
|  |  |  | 5371 | 1 |
|  |  |  | 5400 | 1 |
|  |  | **CC5902** | **-** | **2** |
|  |  |  | 840 | 1 |
|  |  |  | 5336 | 1 |
|  |  | **CC5484/5796** | **-** | **1** |
|  |  |  | 5484 | 1 |
|  |  | **CC702** | **-** | **1** |
|  |  |  | 702 | 1 |
|  |  | **CC499** | **-** | **1** |
|  |  |  | 499 | 1 |
| **15F** | **1** | **-** | **-** | **-** |
|  |  | **Singleton** | **-** | **1** |
|  |  |  | 4156 | 1 |
| **16F** | **6** | **-** | **-** | **-** |
|  |  | **Singleton** | **-** | **3** |
|  |  |  | 5935 | 2 |
|  |  |  | 6116 | 1 |
|  |  | **CC5250/5947** | **-** | **3** |
|  |  |  | 5250 | 2 |
|  |  |  | 5947 | 1 |
| **17F** | **5** | **-** | **-** | **-** |
|  |  | **CC1146** | **-** | **2** |
|  |  |  | 5753 | 2 |
|  |  | **CC848** | **-** | **2** |
|  |  |  | 848 | 2 |
|  |  | **CC4934** | **-** | **1** |
|  |  |  | 5355 | 1 |
| **18C** | **8** | **-** | **-** | **-** |
|  |  | **CC1381** | **-** | **7** |
|  |  |  | 1381 | 7 |
|  |  | **CC28/5067** | **-** | **1** |
|  |  |  | 5068 | 1 |
| **19A** | **9** | **-** | **-** | **-** |
|  |  | **CC847** | **-** | **8** |
|  |  |  | 847 | 7 |
|  |  |  | 5270 | 1 |
|  |  | **Singleton** | **-** | **1** |
|  |  |  | 5372 | 1 |
| **19B** | **7** | **-** | **-** | **-** |
|  |  | **CC5368/7053** | **-** | **6** |
|  |  |  | 5368 | 4 |
|  |  |  | 5391 | 1 |
|  |  |  | 5776 | 1 |
|  |  | **Singleton** | **-** | **1** |
|  |  |  | 6102 | 1 |
| **19F** | **78** | **-** | **-** | **-** |
|  |  | **CC844** | **-** | **37** |
|  |  |  | 844 | 13 |
|  |  |  | 5268 | 1 |
|  |  |  | 5339 | 10 |
|  |  |  | 5367 | 5 |
|  |  |  | 5385 | 1 |
|  |  |  | 5751 | 1 |
|  |  |  | 5763 | 1 |
|  |  |  | 5777 | 1 |
|  |  |  | 5925 | 1 |
|  |  |  | 5929 | 1 |
|  |  |  | 5930 | 1 |
|  |  |  | 5939 | 1 |
|  |  | **CC2715** | **-** | **12** |
|  |  |  | 2715 | 7 |
|  |  |  | 5766 | 1 |
|  |  |  | 5931 | 1 |
|  |  |  | 6088 | 2 |
|  |  |  | 6111 | 1 |
|  |  | **CC2386/5760** | **-** | **6** |
|  |  |  | 2386 | 2 |
|  |  |  | 5331 | 3 |
|  |  |  | 5760 | 1 |
|  |  | **CC347** | **-** | **6** |
|  |  |  | 556 | 2 |
|  |  |  | 5344 | 1 |
|  |  |  | 5754 | 1 |
|  |  |  | 5769 | 1 |
|  |  |  | 6101 | 1 |
|  |  | **Singleton** | **-** | **6** |
|  |  |  | 5341 | 1 |
|  |  |  | 5759 | 2 |
|  |  |  | 5771 | 1 |
|  |  |  | 5942 | 1 |
|  |  |  | 6117 | 1 |
|  |  | **CC849/5343/5351** | **-** | **2** |
|  |  |  | 5343 | 1 |
|  |  |  | 5351 | 1 |
|  |  | **CC5360/5762/6112** | **-** | **2** |
|  |  |  | 5360 | 1 |
|  |  |  | 5762 | 1 |
|  |  | **CC3518** | **-** | **2** |
|  |  |  | 3518 | 2 |
|  |  | **CC848** | **-** | **1** |
|  |  |  | 848 | 1 |
|  |  | **CC988** | **-** | **1** |
|  |  |  | 988 | 1 |
|  |  | **CC703** | **-** | **1** |
|  |  |  | 5943 | 1 |
|  |  | **CC5370** | **-** | **1** |
|  |  |  | 5365 | 1 |
|  |  | **CC5752** | **-** | **1** |
|  |  |  | 843 | 1 |
| **20** | **7** | **-** | **-** | **-** |
|  |  | **CC702** | **-** | **7** |
|  |  |  | 702 | 3 |
|  |  |  | 5350 | 1 |
|  |  |  | 5392 | 2 |
|  |  |  | 5773 | 1 |
| **21** | **5** | **-** | **-** | **-** |
|  |  | **CC177** | **-** | **4** |
|  |  |  | 1145 | 4 |
|  |  | **CC6451** | **-** | **1** |
|  |  |  | 5926 | 1 |
| **23A** | **5** | **-** | **-** | **-** |
|  |  | **Singleton** | **-** | **2** |
|  |  |  | 5366 | 1 |
|  |  |  | 5950 | 1 |
|  |  | **CC4894/6104** | **-** | **1** |
|  |  |  | 4894 | 1 |
|  |  | **CC848** | **-** | **1** |
|  |  |  | 5357 | 1 |
|  |  | **CC844** | **-** | **1** |
|  |  |  | 5339 | 1 |
| **23B** | **16** | **-** | **-** | **-** |
|  |  | **CC5370** | **-** | **11** |
|  |  |  | 5370 | 10 |
|  |  |  | 6099 | 1 |
|  |  | **CC4894/6104** | **-** | **4** |
|  |  |  | 4894 | 3 |
|  |  |  | 6104 | 1 |
|  |  | **CC490** | **-** | **1** |
|  |  |  | 489 | 1 |
| **23F** | **41** | **-** | **-** | **-** |
|  |  | **CC2714** | **-** | **20** |
|  |  |  | 2714 | 17 |
|  |  |  | 5382 | 1 |
|  |  |  | 5750 | 1 |
|  |  |  | 5945 | 1 |
|  |  | **CC988** | **-** | **7** |
|  |  |  | 988 | 6 |
|  |  |  | 2718 | 1 |
|  |  | **CC848** | **-** | **5** |
|  |  |  | 848 | 5 |
|  |  | **Singleton** | **-** | **4** |
|  |  |  | 5388 | 1 |
|  |  |  | 5923 | 1 |
|  |  |  | 6100 | 1 |
|  |  |  | 6113 | 1 |
|  |  | **CC802** | **-** | **2** |
|  |  |  | 802 | 2 |
|  |  | **CC701** | **-** | **1** |
|  |  |  | 2069 | 1 |
|  |  | **CC844** | **-** | **1** |
|  |  |  | 5339 | 1 |
|  |  | **CC2713** | **-** | **1** |
|  |  |  | 5379 | 1 |
| **24F** | **3** | **-** | **-** | **-** |
|  |  | **CC4888/5077** | **-** | **2** |
|  |  |  | 5077 | 2 |
|  |  | **CC2208** | **-** | **1** |
|  |  |  | 2818 | 1 |
| **28F** | **1** | **-** | **-** | **-** |
|  |  | **Singleton** | **-** | **1** |
|  |  |  | 5373 | 1 |
| **29** | **2** | **-** | **-** | **-** |
|  |  | **CC5329/5876** | **-** | **1** |
|  |  |  | 5876 | 1 |
|  |  | **CC1146** | **-** | **1** |
|  |  |  | 5952 | 1 |
| **31** | **1** | **-** | **-** | **-** |
|  |  | **CC3548** | **-** | **1** |
|  |  |  | 444 | 1 |
| **33B** | **2** | **-** | **-** | **-** |
|  |  | **CC5375/5938** | **-** | **1** |
|  |  |  | 5938 | 1 |
|  |  | **CC1146** | **-** | **1** |
|  |  |  | 5932 | 1 |
| **33D** | **2** | **-** | **-** | **-** |
|  |  | **Singleton** | **-** | **1** |
|  |  |  | 5376 | 1 |
|  |  | **CC5333/5901** | **-** | **1** |
|  |  |  | 5333 | 1 |
| **34** | **7** | **-** | **-** | **-** |
|  |  | **CC841/5258** | **-** | **5** |
|  |  |  | 5258 | 5 |
|  |  | **Singleton** | **-** | **1** |
|  |  |  | 5398 | 1 |
|  |  | **CC5934** | **-** | **1** |
|  |  |  | 5934 | 1 |
| **35A** | **5** | **-** | **-** | **-** |
|  |  | **CC5902** | **-** | **5** |
|  |  |  | 840 | 2 |
|  |  |  | 5377 | 1 |
|  |  |  | 5394 | 1 |
|  |  |  | 5772 | 1 |
| **35B** | **19** | **-** | **-** | **-** |
|  |  | **CC1146** | **-** | **16** |
|  |  |  | 1146 | 12 |
|  |  |  | 5396 | 3 |
|  |  |  | 5756 | 1 |
|  |  | **CC5902** | **-** | **1** |
|  |  |  | 840 | 1 |
|  |  | **Singleton** | **-** | **1** |
|  |  |  | 5359 | 1 |
|  |  | **CC176** | **-** | **1** |
|  |  |  | 5358 | 1 |
| **35F** | **2** | **-** | **-** | **-** |
|  |  | **CC5349** | **-** | **2** |
|  |  |  | 5349 | 1 |
|  |  |  | 5353 | 1 |
| **37** | **2** | **-** | **-** | **-** |
|  |  | **CC5250/5947** | **-** | **1** |
|  |  |  | 5250 | 1 |
|  |  | **CC473** | **-** | **1** |
|  |  |  | 5951 | 1 |
| **38** | **2** | **-** | **-** | **-** |
|  |  | **CC5560/6090/6103** | **-** | **1** |
|  |  |  | 6103 | 1 |
|  |  | **CC5360/5762/6112** | **-** | **1** |
|  |  |  | 6112 | 1 |
| **NT** | **2** | **-** | **-** | **-** |
|  |  | **Singleton** | **-** | **1** |
|  |  |  | 5387 | 1 |
|  |  | **CC841/5258** | **-** | **1** |
|  |  |  | 5258 | 1 |
| **Total** | **486** |  |  |  |
